# Professor Bache’s Introductory

**Published:** 1844-01

**Authors:** 


PROFESSOR BACHE'S INTRODUCTORY

This discourse is what it purports to be—strictly an introduction to a course of lectures on Chemistry. It lays down the plan which the lecturer proposes to follow, and discusses, briefly, several topics in the simple, lucid, unambitious style adapted to scientific subjects. The lecture is thoroughly chemical, and is one from which his pupils will derive profit. A very clear statement of Leibig's theory of animal heat and nutrition makes a part of the lecture, and the author, we think, has pointed out one of the premature conclusions, from which it will be admitted by the warmest admirers of the great German Chemist, that his physiological doctrines are by no means free.

Liebig holds that the waste of the organism of animals is repaired by the proteine-compounds of their food—the fibrine, albumen, and caseine, and that gelatine, not being a modification of proteine, takes no part in nutrition, unless it be in that of the gelatinous tissues. The fact upon which he rests this opinion is the starvation of dogs when fed exclusively upon gelatine. But, as Prof. Bache urges, is not the result the same when animals are confined to any single nutritive element? Geese died with the symptoms of starvation, in the experiments of Tiedemann and Gmelin, although they had an ample supply of albumen, the great element of nutrition. In the present state of our knowledge, we are not authorized to say that a substance is not nutritious because it will not, singly, support the life of an animal. The proteine-compounds, containing no gelatine, are adequate to the nourishment of animals. All their organized parts are derived from these compounds, and it follows, that their fibrine or albumen must be transformed by the vital process into the gelatinous tissues. Liebig indicates the mode by which it is done. "But," asks Prof. B., very properly, "why may not the converse operation take place; and if we admit that the vital force can convert proteine into gelatine; why not concede that the same force can change gelatine into proteine, which is equivalent to its change into blood?" We agree with him, that Liebig has here committed an error "in limiting the transforming powers of the digestive and assimilating organs." And we admit it the more willingly, because we have heretofore so freely admitted Liebig's claims to general accuracy, as well as great originality of doctrine. Prof. B. thinks it is conceded by every one, "that chemical changes are incessantly taking place in living beings, whether animal or vegetable; and he agrees with nearly every one who has examined "Liebig's Animal Chemistry," that it has "lifted a corner of the curtain which conceals from our view what is performed on the stage of the vital organism." But he cautions his students against a too ready admission of what he calls its "presumptuous claim," that the chemists have "penetrated behind the scenes, and witnessed nature in the very act of playing her part in the mysterious drama of life." Y.

